# Optimization of Glibenclamide Loaded Thermoresponsive SNEDDS Using Design of Experiment Approach: Paving the Way to Enhance Pharmaceutical Applicability

**DOI:** 10.3390/molecules29215163

**Published:** 2024-10-31

**Authors:** Abdelrahman Y. Sherif, Ehab M. Elzayat, Mohammad A. Altamimi

**Affiliations:** Department of Pharmaceutics, College of Pharmacy, King Saud University, Riyadh 11451, Saudi Arabia; eelzayat1.c@ksu.edu.sa (E.M.E.); maltamimi@ksu.edu.sa (M.A.A.)

**Keywords:** Thermoresponsive SNEDDS, Poloxamer 188, propylene glycol, glibenclamide, in vitro dissolution, DoE

## Abstract

Thermoresponsive self-nanoemulsifying drug delivery systems (T-SNEDDS) offer a promising solution to the limitations of conventional SNEDDS formulations. Liquid SNEDDS are expected to enhance drug solubility; however, they are susceptible to leakage during storage. Even though solid SNEDDS offers a solution to this storage instability, they introduce new challenges, namely increased total dosage and potential for drug trapping within the formulation. The invented T-SNEDDS was used to overcome these limitations and improve the dissolution of glibenclamide (GBC). Solubility and transmittance studies were performed to select a suitable oil and surfactant. Design of Experiments (DoE) software was used to study the impact of propylene glycol and Poloxamer 188 concentrations on measured responses (liquefying temperature, liquefying time, and GBC solubility). The optimized formulation was subjected to an in vitro dissolution study. The optimized T-SNEDDS consisted of Kolliphor EL and Imwitor 308 as surfactants and oil. The optimized propylene glycol and Poloxamer 188 concentrations were 13.7 and 7.9% *w*/*w*, respectively. It exhibited a liquefying temperature of 35.0 °C, a liquefying time of 119 s, and a GBC solubility of 5.51 mg/g. In vitro dissolution study showed that optimized T-SNEDDS exhibited 98.8% dissolution efficiency compared with 2.5% for raw drugs. This study presents a promising approach to enhance pharmaceutical applicability by resolving the limitations of traditional SNEDDS.

## 1. Introduction

Self-nano-emulsifying drug delivery systems (SNEDDS) have been widely used to boost the oral bioavailability of lipophilic drugs [1]. They consist of a homogenous blend of surfactant, co-surfactant, and oil that can form a nanoemulsion within the gastrointestinal tract (GIT) following exposure to agitation by peristaltic movement [2,3]. The dispersed nanoemulsion facilitates the solubilization of drugs and enhances their oral bioavailability [4]. However, liquid SNEDDS suffer from a propensity to leak from soft gelatin capsules, which limits their application as a pharmaceutical dosage form [5,6,7].

Consequently, Solid SNEDDS have been invented to overcome the limitations of liquid SNEDDS using various technologies, including lyophilization [8], hot melt extrusion [9], spray drying [10], and fluid bed coating [6]. However, the associated high cost of these technologies owing to the multistep processes during production and expensive instruments limit their application. Even though adsorption onto porous materials overcomes these limitations, drug trapping and high total dosage hinder its application [11,12]. Therefore, an alternative approach is still required to address the limitations of traditional forms of SNEDDS.

Herein, a novel thermoresponsive SNEDDS (T-SNEDDS) formulation has been invented in response to this demand. This innovative formulation combines the advantages of a low dosage of liquid SNEDDS with the leak-prevention properties of solid SNEDDS. The incorporation of Poloxamer 188 presents an essential rule, which enables the formulation to remain solid at room temperature, prevent leakage during storage, and convert to a liquid state at body temperature, facilitating optimal drug release and absorption. Propylene glycol is incorporated into the SNEDDS formulation as a cosurfactant for Poloxamer 188 and modulates SNEDDS transition from a solid to a liquid state to achieve this thermoresponsive behavior.

To investigate the ability of T-SNEDDS to remain solid at room temperature while retaining the advantages of enhancing drug dissolution, glibenclamide (GBC) was utilized as a model drug. It is widely prescribed by physicians owing to its reported potency and long duration of action [13]. It belongs to sulfonylureas, which are widely used to treat patients diagnosed with diabetes mellitus (DM), particularly type II. They reduce blood glucose levels following pancreatic beta cell stimulation, which results in a pronounced increment in insulin secretion [14].

Even though GBC showed promising outcomes in clinical settings during the treatment of diabetes, it exhibits poor oral bioavailability (approximately 45%) due to its low aqueous solubility [15]. Previous studies were conducted to improve the poor aqueous solubility and dissolution associated with GBC [16,17,18,19]. However, none of these studies address the limitation of formulation leakage. Therefore, GBC is a good candidate for preparing T-SNEDDS to enhance poor aqueous dissolution and prevent formulation leakage during storage.

To select the optimum T-SNEDDS formulation, Design of Experiments (DoE) software was utilized. This systematic approach simultaneously studies the interaction of multiple factors, which gives a precise indication of the impact of independent factors on measured response factors [20,21]. Using DoE software, the influence of propylene glycol and Poloxamer 188 concentrations on the liquefying temperature, liquefying time, and GBC solubility of prepared T-SNEDDS formulations was studied.

This study aims to prepare optimized T-SNEDDS as a potential alternative to conventional SNEDDS formulations. The optimization process was performed in two stages. First, oil was selected based on solubility to achieve maximum drug loading, while surfactant was selected based on an emulsification study to ensure the ability to form nanoemulsion droplets with the selected oil. Second, the influence of propylene glycol and Poloxamer 188 concentrations on the thermoresponsive behavior of T-SNEDDS was investigated using Design-Expert^®^ software. The suggested optimized formulation was then prepared and subjected to pharmaceutical assessment, including particle size analysis and in vitro dissolution.

## 2. Results and Discussion

### 2.1. Selection of Oil

**Figure 1:**
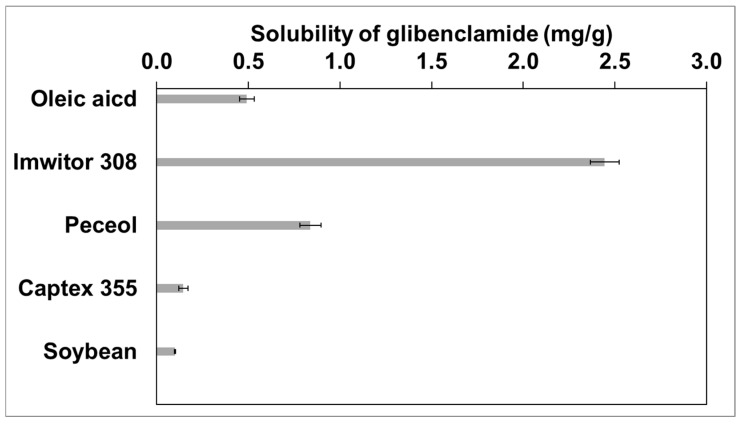
Solubility of glibenclamide in different oils.

The solubility of GBC within different types of oils was studied to select the lipid phase during the preparation of T-SNEDDS. The oils were carefully chosen to represent diverse chemical classes: long-chain triglycerides (soybean oil), medium-chain triglycerides (Captex 355), long-chain monoglycerides (Peceol), short-chain monoglycerides (Imwitor 308), and free fatty acids (oleic acid). This strategy aimed to assess the impact of oils with different esterification degrees and chain lengths on their solubilization capacity. Figure 1 showed that Captex 355 and soybean oils (triglycerides) have a lower ability to solubilize GBC, with values of 0.15 ± 0.02 and 0.10 ± 0.00 mg/g, respectively. Oleic acid (free fatty acids) increased GBC’s solubility to 0.49 ± 0.04 mg/g. Moreover, higher GBC solubility was detected in Peceol and Imwitor 308 oil (monoglycerides), with values of 0.84 ± 0.06 and 2.44 ± 0.08 mg/g, respectively. Consequently, Imwitor 308 was selected to prepare T-SNEDDS based on its measured high drug solubility. This could enable the incorporation of GBC within dispersed nanoemulsions inside the gastrointestinal tract and avoid drug precipitation [22]. Furthermore, solubilization of GBC will ensure high drug bioavailability by maintaining a noteworthy concentration gradient driving force between GIT and systemic circulation [23].

### 2.2. Selection of Surfactant

**Figure 2:**
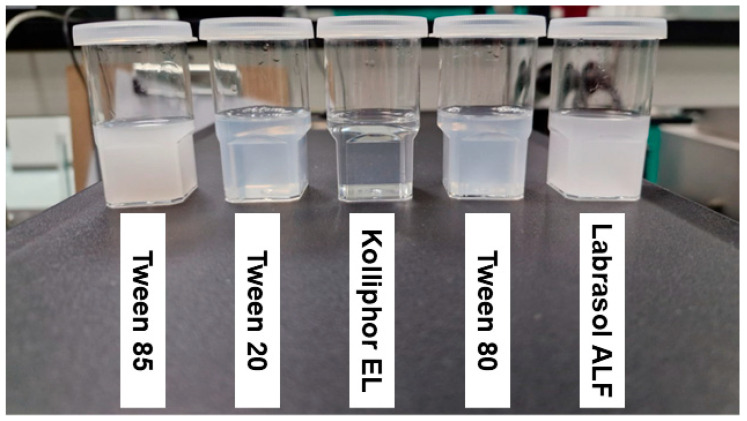
Physical appearance of a dispersed mixture consisting of Imwitor 308 and different types of surfactants.

Transmittance measurement was performed to select the optimum surfactant during the preparation of T-SNEDDS. The prepared mixtures (surfactant and oil) were dispersed, showing their physical appearance in Figure 2. In addition, transmittance percentages were measured to give a numerical value for each dispersion and presented in Table 1. It is clear from the images that the mixture containing tween 85 and labrasol ALF produced a milky dispersion system that agrees with the low transmittance value of less than 5%. The dispersion of the mixture comprising tween 80 and tween 20 produced a pale white dispersion system with a transmittance value of about 60%. Finally, Kolliphor EL enhanced the dispersion of Imwitor 308 oil, and the dispersed system appeared clear with a transmittance value of about 99%. The measured high transmittance value of Kolliphor EL and Imwitor 308 mixture confirms its dispersion in the nanosized range [24].

### 2.3. Selection of Cosurfactant

**Figure 3:**
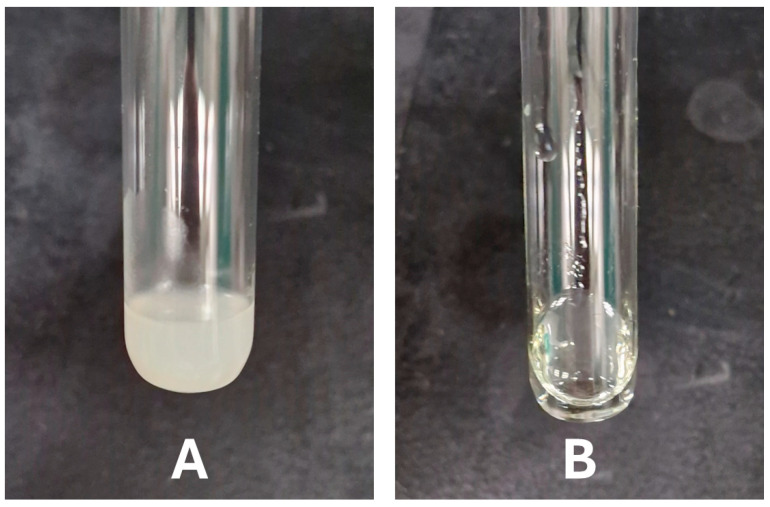
Physical appearance of thermoresponsive SNEDDS formulation: (**A**) Solid state at room temperature (25 °C), and (**B**) Liquid state at body temperature (37 °C).

Thermoresponsive polymer (Poloxamer 188) was selected as a solidifying agent to prepare the T-SNEDDS formulation. Poloxamer 188 at a concentration of 10% *w*/*w* was subjected to mixing with Kolliphor EL and Imwitor 308 for one day, and the polymer failed to dissolve. On the other hand, Poloxamer 188 could dissolve in propylene glycol, which could be attributed to the formation of hydrogen bonding between them. The detected insolubility of Poloxamer 188 in Kolliphor EL and Imwitor 308 could be ascribed to complex molecular interaction. Although Kolliphor EL contains hydroxyl groups, its complex structure sterically hinders the formation of hydrogen bonds with Poloxamer 188. Furthermore, Imwitor 308 has a free hydroxyl group and is considered a lipophilic molecule owing to the presence of lipophilic caprylic acid fatty acid. Figure 3A shows T-SNEDDS remains in a solid at room temperature 25 °C, while it transitions to a liquid state when exposed to body temperature 37 °C (Figure 3B). This thermoresponsive property enhances the formulation’s stability during storage and facilitates its conversion to a liquid form upon administration. Therefore, it was selected as a cosurfactant to solubilize Poloxamer 188 within T-SNEDDS.

### 2.4. Effect of Independent Variables on the Responses

**Figure 4:**
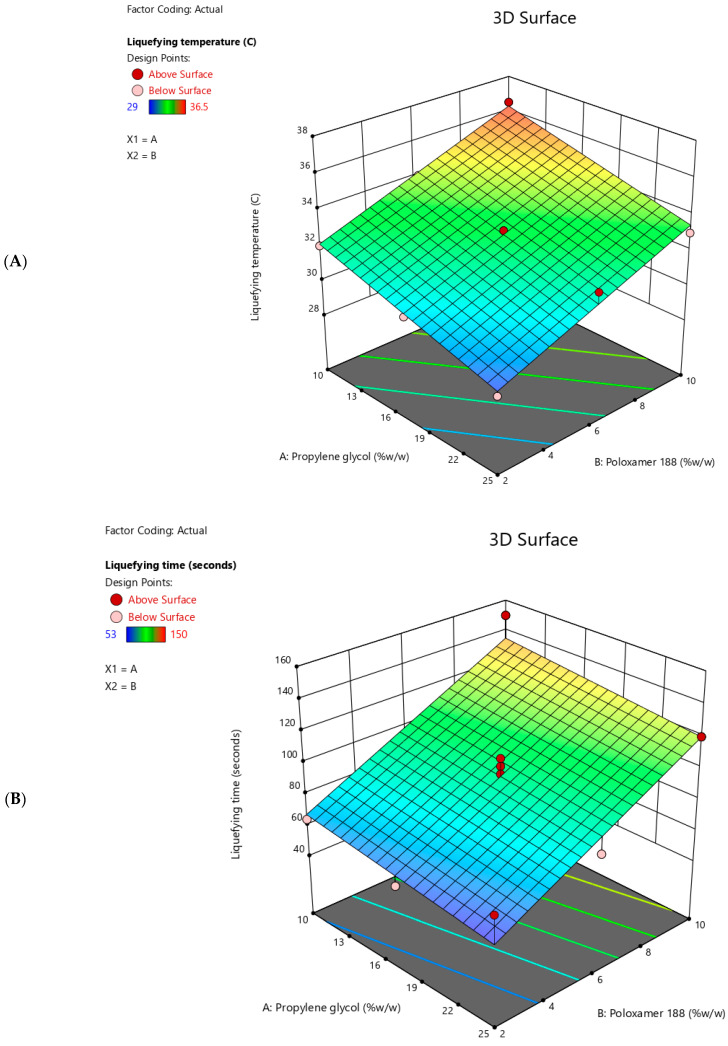

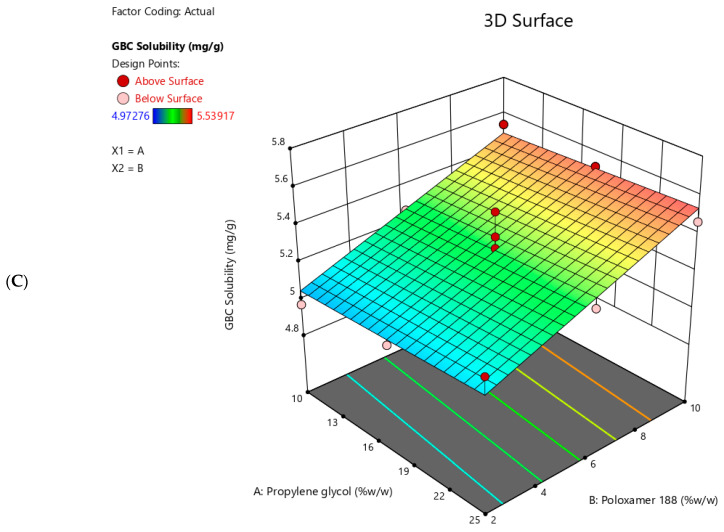
Three-dimensional surface plots showing the influence of propylene glycol and Poloxamer 188 concentrations on (**A**) liquefying temperature, (**B**) liquefying time, and (**C**) GBC Solubility of the thermoresponsive SNEDDS formulations.

Table 2 shows the measured responses of the prepared T-SNEDDS, including liquefying temperature, liquefying time, and GBC solubility. The DOE software was utilized to examine the influence of Poloxamer-188 and propylene glycol concentrations on the measured responses using various mathematical models, including linear, 2FI, quadratic, and cubic. The best-fitting model was selected based on statistical parameters. The selected model showed no significant lack of fit (*p* > 0.05), indicating good model adequacy. Table 3 summarizes the selected models for each response based on ANOVA analysis, while Figure 4 shows the 3D surface plots for the measured responses. The impact of Poloxamer-188 and propylene glycol concentrations on the studied responses was discussed individually.

### 2.5. Liquefying Temperature

**Figure 5:**
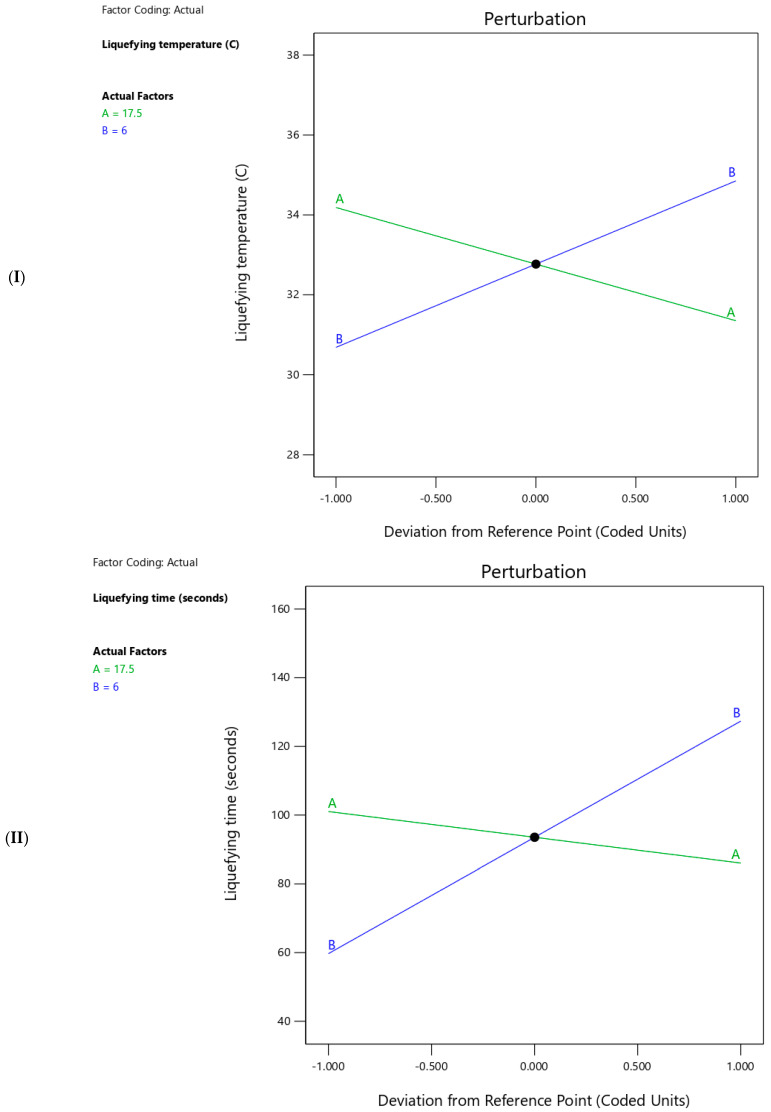

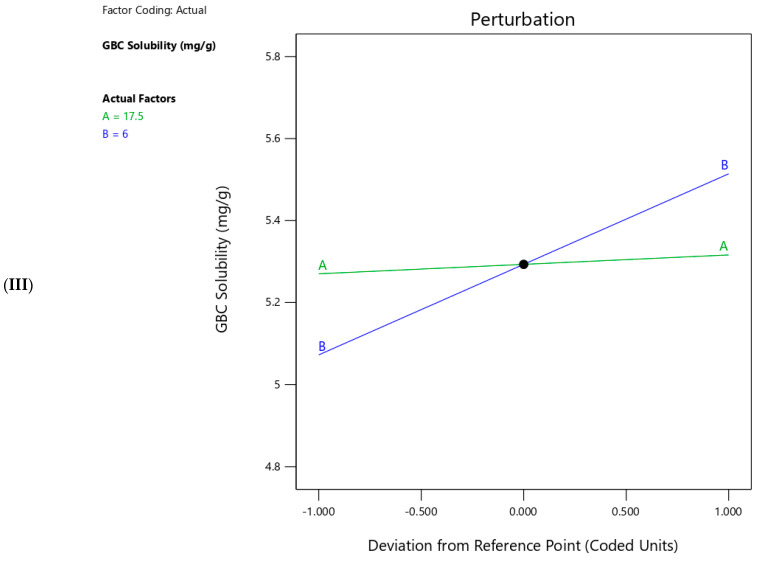
Perturbation plots illustrate the effects of (A) propylene glycol (green line) and (B) Poloxamer 188 (Blue line) on (**I**) liquefying temperature, (**II**) liquefying time, and (**III**) GBC Solubility of the in-situ liquefying SNEDDS formulations. The x-axis represents the deviation from the reference point in coded units, while the y-axis shows the response value. Green lines represent propylene glycol, and blue lines represent Poloxamer 188.

The prepared thermoresponsive SNEDDS had liquefying temperatures ranging from 29 to 36.5 °C (Table 2). Statistical analysis showed that increasing the concentration of propylene glycol resulted in a significant (<0.0001) reduction in the liquefying temperature of thermoresponsive SNEDDS (Table 4). On the contrary, increasing the concentration of Poloxamer 188 resulted in a significant (<0.0001) increment in the liquefying temperature of thermoresponsive SNEDDS (Table 4). The steepness of the lines (Figure 5I) aligns with statistical analysis, which indicates the sensitivity of both responses to changes in each factor. In addition, the liquefying temperature of the thermoresponsive SNEDDS could be expected utilizing Equation (1):

Liquefying temperature = 32.95 − 0.19 ∗ Propylene glycol (% *w*/*w*) + 0.52 ∗ Poloxamer 188 (% *w*/*w*) (1)

The thermoresponsive behavior of the prepared formulations can be attributed to the complex interactions between propylene glycol and Poloxamer 188, which is in agreement with a previously reported study [25]. At lower temperatures, the hydroxyl groups of propylene glycol could form hydrogen bonding with terminal hydroxyl groups or oxygen atoms in the intra-polyether part of Poloxamer 188. This agrees with previous studies, which showed that Poloxamer forms hydrogen bonds with the hydroxyl groups of polyacrylic acid and Carboxymethyl Pullulan, respectively [26,27]. Moreover, Poloxamer could create two types of bonding between the polymer units: intermolecular hydrogen bonding and Van der Waals forces. This further supports our study, which showed that intermolecular hydrogen bonds could be formed between terminal hydroxyl groups of long hydrocarbon chains [28]. Moreover, dipole interactions between Poloxamer units could be generated by electrons withdrawing oxygen atoms within polymer chains [29]. The predicted complex crosslinking between propylene glycol and Poloxamer 188 could be the reason for solidification. On the contrary, increasing temperatures break these bonds and form a solubilized micellular structure [30]. Therefore, T-SNEDDS is converted from a solid to a liquid state.

Increasing propylene glycol reduces the liquefying temperature, resulting in a loose crosslinking structure. This could be ascribed to propylene glycol’s small molecular size. Therefore, increasing propylene glycol concentration separates Poloxamer 188 units from each other and prevents the formation of complex bridging bonds between polymer units. On the contrary, increasing the Poloxamer concentration formed a rigid matrix with a cross-linking solid structure. This aligns with the principle that increased polymer concentration leads to the formation of rigid structures [31]. Therefore, higher energy is required to break it down and form a soluble micellar structure.

### 2.6. Liquefying Time

The prepared thermoresponsive SNEDDS had liquefying times ranging from 53 to 150 s (Table 2). Statistical analysis showed that increasing the concentration of propylene glycol rendered insignificant (*p*-value = 0.1188) the reduction in the liquefying time of thermoresponsive SNEDDS (Table 4). In contrast, increasing the concentration of Poloxamer 188 resulted in a significant (<0.0001) increment in the liquefying time of thermoresponsive SNEDDS (Table 4). The slope of the line (B) (Figure 5II) aligns with statistical analysis, which indicates the sensitivity of liquefying time to changes in Poloxamer 188 concentration alone. In addition, the liquefying time of the thermoresponsive SNEDDS could be expected utilizing Equation (2) as follows:

Liquefying time = 60.29 − 1.00 ∗ Propylene glycol (% *w*/*w*) + 8.46 ∗ Poloxamer 188 (% *w*/*w*) (2)

The obtained results are consistent with liquefying temperature. The observed significant influence in Poloxamer 188 could result from forming a rigid structure while increasing Poloxamer concentration. Moreover, this is consistent with the reported thermoresponsive behavior of poloxamer [32]. On the other hand, the insignificant effect of propylene glycol is attributed to the measuring temperature of 37 °C, which is above the liquefying temperature for all formulations. Therefore, the micellization of poloxamer is mainly driven by its concentration rather than propylene glycol concentration. This agreed with a previously reported study by Alexandridis et al., who found that the conversion of poloxamer from a monomeric state to micellar form is easily reached with increasing temperature [33].

### 2.7. GBC Solubility

The prepared thermoresponsive SNEDDS had GBC solubility ranging from 4.97 to 5.54 mg/g (Table 2). Statistical analysis showed that increasing the concentration of propylene glycol rendered insignificant (*p*-value = 0.5792) the reduction in the GBC solubility of thermoresponsive SNEDDS (Table 4). In contrast, increasing the concentration of Poloxamer 188 resulted in a significant (<0.0001) increment in the GBC solubility of thermoresponsive SNEDDS (Table 4). The slope of the line (B) (Figure 5III) aligns with statistical analysis, which indicates the sensitivity of GBC solubility to changes in Poloxamer 188 concentration rather than propylene glycol concentration. In addition, the liquefying time of the thermoresponsive SNEDDS could be expected utilizing Equation (3) as follows:

GBC Solubility = 4.91 + 0.003 ∗ Propylene glycol (% *w*/*w*) + 0.06 ∗ Poloxamer 188 (% *w*/*w*) (3)

The significant positive effect of Poloxamer 188 on GBC solubility demonstrates its effectiveness in enhancing drug solubilization. The observed significant effect of poloxamer could be attributed to its amphiphilic nature and its ability to solubilize hydrophobic drugs, which agreed with previously reported studies [34]. However, propylene glycol’s lack of a significant effect on the solubility of GBC is noteworthy. There is no scientific rationale for this observation. However, further investigation is required to address the complexity of bonding within the T-SNEDDS formulation.

### 2.8. Optimization of Thermoresponsive SNEDDS

**Figure 6:**
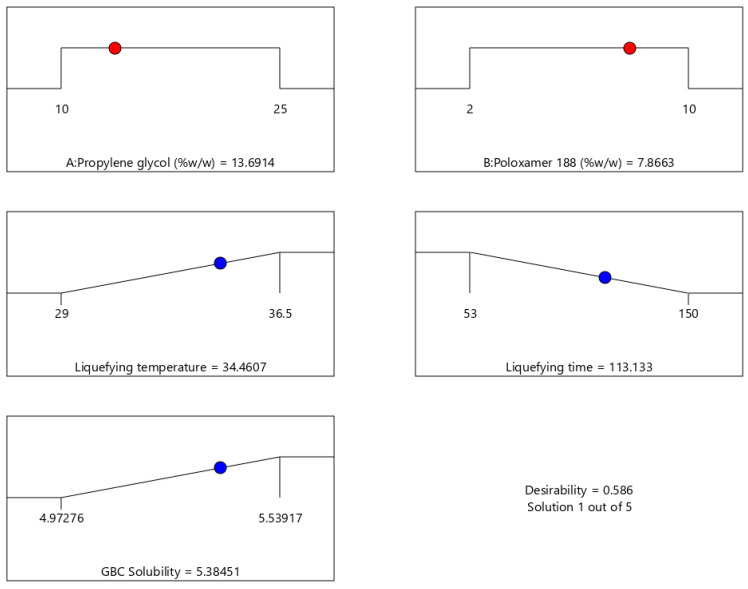
Optimization results for thermoresponsive SNEDDS formulation. Each subplot displays the range of values explored for each factor or response, with the blue dot indicating the optimized value within that range.

The optimized T-SNEDDS was chosen based on maximum liquefying temperature and GBC solubility while minimizing liquefying time. The optimization suggested a thermoresponsive SNEDDS formulation comprising 13.7 and 7.9% *w*/*w* propylene glycol and Poloxamer 188, respectively. The proposed optimized formulation showed considerable desirability, as shown in Figure 6. The liquefying temperature of 34.5 °C is far from room temperature (25 °C) and close to body temperature (37 °C). This is required to avoid premature liquefaction during storage before administration and to ensure its transition from solid to liquid upon administration. The liquefying time of 113 s will ensure rapid liquefaction, which promotes rapid drug dissolution in vivo. The GBC solubility of 5.38 mg/g indicates proper drug loading, which reduces the total dosage of the formulation.

The suggested optimized thermoresponsive SNEDDS was prepared to determine the actual values of measured responses. This ensures the validation, accuracy, and reliability of the model suggested by the Design of Experiments software. Table 5 shows the predicted mean value for the measured responses (liquefying temperature, liquefying time, and GBC solubility) against the actual mean values. The results showed that all actual mean values fall within the 95% prediction interval, indicating the developed model’s remarkable power.

### 2.9. Particle Size Measurement

The optimized T-SNEDDS formulation (drug-free) was prepared for pharmaceutical assessment. GBC was mixed with the optimized T-SNEDDS formulation to prepare a drug-loaded formulation. Both drug-free and drug-loaded T-SNEDDS formulations were subjected to particle size analysis. The present results revealed that both formulations dispersed in the nanosize range, with values of 23.8 ± 0.7 and 29.5 ± 1.2 nm, respectively. The observed increase in particle size could be attributed to incorporating GBC with the lipid core of the dispersed nanoemulsion. Moreover, the dispersion of the optimized formulation in the nanosize range indicates its potential to enhance drug bioavailability [35].

### 2.10. In Vitro Dissolution

**Figure 7:**
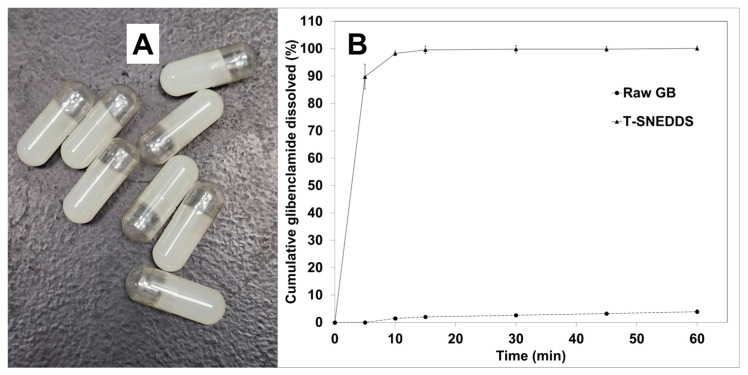
(**A**) Hard gelatin capsules containing thermoresponsive SNEDDS formulation stored at room temperature. (**B**) In vitro dissolution profiles of raw GBC and GBC-loaded T-SNEDDS.

The optimized formulation was placed within a hard gelatin capsule, as shown in Figure 7A. It is clear from the images that the prepared formulation is solidified with no risk of formulation leakage. Figure 7B shows the dissolution profile of GBC from hard gelatin capsules filled with raw drug and T-SNEDDS formulation. The obtained results revealed that pure GBC’s dissolution efficiency was 2.5%. The optimized T-SNEDDS formulation increased dissolution efficiency 39 times with a value of 98.8%. The present results showed that the prepared formulation could enhance the bioavailability of orally administered GBC owing to the observed enhancement in the drug dissolution profile [36,37].

### 2.11. Future Prospective

Even though the prepared optimized T-SNEDDS improved the dissolution of GBC and resolved the limitation of traditional forms of liquid SNEDDS, further studies are still required to study drug permeability. However, it has been reported that SNEDDS formulations were able to improve drug permeability through solubilization and the P-glycoprotein inhibition effect of their excipients. Moreover, Poloxamer showed promising results in enhancing drug permeability through the modulation of tight junctions. Therefore, it is expected that combining both SNEDDS and Poloxamer could augment drug permeability. However, further studies are required to confirm this issue.

Another issue is the impact of different grades of Poloxamer on the T-SNEDDS, which should be addressed. This could include Poloxamer 407, Poloxamer 237, and Poloxamer 338, which vary in molecular weights, hydrophilic-lipophilic balance (HLB) values, and critical micelle concentrations (CMC). This investigation could help identify the optimal thermoresponsive polymer to be used during the preparation of T-SNEDDS for pharmaceutical applications.

## 3. Materials and Methods

### 3.1. Materials

Glibenclamide was acquired from Saudi Pharmaceutical Industries and Medical Appliances Corp. (Qassim, Saudi Arabia). Oleic acid and Imwitor-308 oil were provided by Avonchem (Cheshire, UK) and Sasol Germany GmbH (Werk, Witten, Germany), respectively. Peceol (oil) was supplied by Gattefosse (Saint-Priest, France). Soybean oil and Captex 355 EP/NF were acquired from John L. Seaton & Co., Ltd., Croda International Plc. (East Yorkshire, UK) and Abitec Corporation (Janesville, WI, USA), respectively. Kolliphor EL, Tween-80, and Labrasol ALF (LB) were purchased from BASF (Ludwigshafen, Germany), Loba Chemie (Mumbai, India), and Gattefosse (Saint-Priest, France), respectively. In addition, Tween 20 and Tween 85 were donated by BDH (Poole, UK) and Merck-Schuchardt OHG (Hohenbrunn, Germany), respectively. Propylene glycol and polyethylene glycol 400 were purchased from Winlab Laboratory (Leicestershire, UK) and BASF (Ludwigshafen, Germany), respectively. Poloxamer 188 (average molecular weight ~7680–9510 g/mol) was obtained from Sigma Aldrich (St. Louis, MO, USA).

### 3.2. Ultra Performance Liquid Chromatography (UPLC) Method for Drug Analysis

GBC concentrations in the samples were analyzed using an Ultimate 3000 UPLC system (Thermo Scientific, Bedford, MA, USA) that incorporated a quadratic pump, an automatic sampler, a column chamber, and a Photodiode Array (PDA) detector. The analysis employed an Acquity UPLC BEH C18 column (2.1 × 50 mm, 1.7 μm), through which a mobile phase flowed at 0.3 mL/min. This mobile phase comprised 46.9% acetonitrile and 53.1% of a 0.1% formic acid solution. The column was kept at a constant temperature of 38.8 °C. The concentration of GB in the samples was precisely determined using the PDA detector set at a wavelength of 228 nm. The system was controlled through Chromeleon software version 5 for data acquisition and analysis.

### 3.3. Selection of Oil

GBC solubility within different types of oils was studied to select the lipid phase of SNEDDS formulation. The excess amount of GBC was mixed with each oil using a magnetic stirrer for one day at 1000 rpm. Afterward, the mixture was centrifuged at 14,000 rpm for 5 min to precipitate the undissolved drug. The concentration of GBC in the supernatant was determined utilizing UPLC following appropriate dilution using an organic solvent (acetonitrile).

### 3.4. Selection of Surfactant

Various types of surfactants were subjected to the emulsification study to optimize SNEDDS components. Briefly, Imwitor 308 and surfactant were mixed in an equivalent amount and then heated to facilitate the formulation of a uniform system. The prepared mixture was diluted with distilled water (1:100) to promote the formation of nanoemulsion droplets. The transmittance of the dispersed system was determined using a UV-Vis spectrophotometer Ultrospec 2100 Pro, Amersham Biosciences (Piscataway, NJ, USA) at 638 nm. Distilled water was used as blank during absorbance measurement [38].

### 3.5. Selection of Cosurfactant

The solubility of Poloxamer 188 in various types of cosurfactants (propylene glycol and polyethylene glycol 400) was studied to select the optimum cosurfactant during the preparation of T-SNEDDS. Poloxamer 188 was mixed with cosurfactant to prepare a 10% *w*/*w* concentration. The mixture was stirred for one day at 1000 rpm.

### 3.6. Design of Experiments

In the present study, the optimization process involved two stages. In the first stage, oil (Imwitor 308) was selected based on a solubility study to achieve maximum drug loading within the formulation. Moreover, Kolliphor EL was chosen as the surfactant based on its emulsification efficiency based on measured transmittance value. In the second stage, a cosurfactant (propylene glycol) and polymer (Poloxamer 188) were selected to induce the thermoresponsive behavior of T-SNEDDS formulation. Therefore, this study was designed to investigate the impact of their concentrations on the measured responses. Therefore, the ratio of surfactant and oil (2:1) was kept constant for all formulations to avoid any possible influence in the measured responses. Design-Expert^®^ software (version 13, Stat-Ease Inc., Minneapolis, MN, USA) was used to achieve this purpose.

Design of Experiments (DoE) software using response surface methodology (RSM) was utilized to optimize the T-SNEDDS formulation. The Central Composite Face-Centered (CCF) design was selected precisely because it studies the impact of selected factors at three levels, which provides good prediction capability and assists in efficiently predicting quadratic effects with fewer experimental runs. It consists of four factorial points, four axial points, and five replicated center points, totaling 13 experimental runs. Two independent variables were studied: propylene glycol concentration (X1: 10–25% *w*/*w*) and Poloxamer 188 concentration (X2: 2–10% *w*/*w*). Three response variables were evaluated, including Y1: Liquefying temperature (°C), Y2: Liquefying time (seconds), and Y3: GBC solubility (mg/g). The model was selected based on analysis of variance (ANOVA), lack of fit tests, R^2^ values (R^2^ > 0.9 considered acceptable), comparison of predicted vs. adjusted R^2^, and adequate precision (signal-to-noise ratio > 4 desired). The model was selected based on the sum of squares, lack of fit tests, and statistical analysis in terms of R^2^ values (adjusted and predicted), adequate precision (signal-to-noise ratio), and ANOVA (*p* < 0.05 considered significant).

### 3.7. Preparation of SNEDDS Formulation

The suggested formulations by the Design of Experiments software presented in Table 6 were prepared as follows: a 2:1 mixture of surfactant (Kolliphor-EL) and oil (Imwitor 308) was mixed. Then, propylene glycol and Poloxamer 188 were mixed with this mixture as per Table 6. The prepared formulations were kept in an incubator at 40 °C for two hours to facilitate the solubilization of Poloxamer 188.

### 3.8. Determination of Liquefying Temperature

The suggested 13 and optimized T-SNEDDS formulations were kept in test tubes and left for an hour to facilitate the transition to a solid state. Later, the water bath temperature was set at 25 ± 0.5 °C, and racks holding test tubes were placed in the bath for 3 min for equilibration. Then, the test tubes were inspected visually to determine whether liquefaction had occurred. After that, the temperature was raised by 0.5 ± 0.1 °C, and the formulations were subjected to a similar procedure until the liquefying temperature for all formulations was determined.

### 3.9. Determination of Liquefying Time

The water bath was set at body temperature (37 ± 0.1 °C), and liquefying time for each formulation was determined separately. The test tube was placed in a water bath, and liquefying time was determined once the formulation was completely converted from a solid to a liquid state.

### 3.10. Determination of GBC Solubility

The solubility of GBC within prepared T-SNEDDS was determined by subjection of a mixture of an excess amount of GBC at a given temperature and formulation to stirring at 1000 rpm at a controlled room temperature (23 ± 2 °C). After one day, the mixture was centrifuged at 14,000 rpm for 5 min to precipitate the undissolved drug. The concentration of GBC in the supernatant was determined utilizing UPLC following appropriate dilution using an organic solvent (acetonitrile).

### 3.11. Particle Size Measurement

The particle size of prepared drug-free and drug-loaded T-SNEDDS was measured using the Zetasizer instrument Model ZEN3600, Malvern Instruments Co. (Worcestershire, UK) [39]. A dispersed nanoemulsion system was attained by diluting the prepared formulation using distilled water (1: 1000) and mixing for 5 min using a magnetic stirrer. After that, each sample was placed inside a Zetasizer instrument and allowed for equilibration at 25 °C.

### 3.12. In Vitro Dissolution

The in vitro dissolution study was performed using dissolution apparatus Type II (LOGAN Inst. Corp., Somerset, NJ, USA). A drug-loaded formulation was prepared with 4 mg/g loading based on approximately 80% drug solubility. An equivalent amount of formulation containing GBC (2.5 mg) and row drug was placed inside a hard gelatin capsule. A sinker surrounded the capsules to prevent them from floating during the experiment. Before the experiment, 900 mL of dissolution medium (phosphate buffer, pH 6.8) was preheated at 37 ± 0.5 °C. During the experiment, the paddle’s speed was set at 50 rpm. At predetermined intervals, samples were taken from the medium using a 10-micron filter connected to the syringe. Drug quantification in the samples was determined using the UPLC method described in Section 3.2.

## 4. Conclusions

The present study showed that a prepared thermoresponsive optimized formulation could potentially enhance the pharmaceutical applicability of SNEDDS as a marketed dosage form. The results showed that propylene glycol and Poloxamer 188 concentrations significantly affect the liquefying temperature, while Poloxamer 188 concentrations also significantly affected liquefying time and GBC solubility. The optimized formulation showed desirable characteristics, with a liquefying temperature close to body temperature, rapid liquefying time, and enhanced GBC solubility. The in vitro dissolution study showed that optimized T-SNEDDS significantly enhanced drug dissolution compared to raw GBC.

## Figures and Tables

**Table 1:** The calculated transmittance values for prepared SNEDDS formulations.

Type of Surfactant in Mixture	Transmittance (%)	Physical Appearance
Tween 85	0.37 ± 0.00	Milky
Tween 20	60.23 ± 0.09	Pale white
Kolliphor EL	98.62 ± 0.00	Clear
Tween 80	57.28 ± 0.03	Pale white
Labrasol ALF	1.22 ± 0.05	Milky

**Table 2:** Measured responses of the prepared in situ liquefying SNEDDS formulations.

Run	In Situ Liquefying Temperature (°C)	In Situ Liquefying Time (Seconds)	Glibenclamide Solubility (mg/gm)
1	33	105	5.30
2	33	84	5.15
3	33	120	5.47
4	33	100	5.24
5	32	75	5.26
6	30.5	53	5.04
7	33	84	5.37
8	34	96	5.26
9	32	64	4.97
10	32.5	95	5.50
11	29	70	5.19
12	34.5	120	5.52
13	36.5	150	5.54

**Table 3:** ANOVA analysis of the measured responses for the selected models.

Response	Selected Model	Degree of Freedom	Adjusted R2	Predicted R2	F-Value	*p*-Value
Liquefying temperature	Linear	2	0.9626	0.9404	155.52	<0.0001
Liquefying time	Linear	2	0.8337	0.7073	31.07	<0.0001
GBC solubility	Linear	2	0.7047	0.6102	15.32	0.0009

**Table 4:** ANOVA summary presenting the significance (*p*-values) of independent variables on measured responses (Liquefying temperature, liquefying time, and GBC solubility).

Response	Propylene Glycol Concentration	Poloxamer 188 Concentration
Liquefying temperature	<0.0001	<0.0001
Liquefying time	0.1188	<0.0001
GBC solubility	0.5792	0.0003

**Table 5:** Comparison of Predicted and Observed Values for Key Parameters of thermoresponsive SNEDDS Formulations.

Response	n	Predicted Mean	Actual Mean
Liquefying temperature (°C)	3	34.46	35
Liquefying time (s)	3	113.13	119
GBC Solubility (mg/g)	3	5.38	5.51

**Table 6:** Suggested 13 runs by Design of Experiments for Thermoresponsive Self-Nanoemulsifying Drug Delivery System (T-SNEDDS).

Std	Run	Factor 1: Propylene Glycol	Factor 2: Poloxamer 188	Kolliphor EL (mg)	Imwitor 308 (mg)	Propylene Glycol (mg)	Poloxamer 188 (mg)
13	1	17.5	6	1785	893	613	210
11	2	17.5	6	1785	893	613	210
4	3	25	10	1517	758	875	350
9	4	17.5	6	1785	893	613	210
6	5	25	6	1610	805	875	210
7	6	17.5	2	1878	939	613	70
12	7	17.5	6	1785	893	613	210
5	8	10	6	1960	980	350	210
1	9	10	2	2053	1027	350	70
10	10	17.5	6	1785	893	613	210
2	11	25	2	1703	852	875	70
8	12	17.5	10	1692	846	613	350
3	13	10	10	1867	933	350	350

## Data Availability

Data are contained within the article.
